# Agrivoltaics for Rural Development: Energy Security, Crop Diversification, and Sustainability on Mexican Rainfed Maize Farms

**DOI:** 10.1002/gch2.70133

**Published:** 2026-07-23

**Authors:** Shahin Rasooli, Shahrzad Farhoodi, Catalina Spataru, Pabel Antonio Cervantes‐Avilés, Carlos Alberto Huerta‐Aguilar

**Affiliations:** ^1^ School of Engineering and Sciences Tecnológico de Monterrey Puebla Mexico; ^2^ Energy Institute University College London (UCL) London UK

**Keywords:** agrivoltaics, rural electrification, rural development, sustainable development, water‐energy‐food nexus

## Abstract

Resource availability, poverty, and underdevelopment are bottlenecks of modernization in rural communities. This study proposes a rural electrification and development strategy through deploying agrivoltaics (AV) systems on rainfed maize farms in rural Mexico. As an alternative to maize monoculture, we proposed lettuce, tomato, spinach, and potato in a crop rotation system. The strategy was evaluated through techno‐economic and sustainability assessments under different adoption scenarios (5%, 10%, and 15% conversion rates of rainfed maize fields) and household energy demand profiles. Under the baseline energy demand growth rate, the results demonstrate that AV adoption can substantially enhance the net present value (NPV) of the studied municipality. Specifically, compared to the business‐as‐usual scenario's value of 86.7 million MXN, NPV increases to 162.4, 230.0, and 297.4 million MXN under the 5%, 10%, and 15% AV adoption scenarios, respectively, driven by the combined revenues generated from power sales and agricultural production. The photovoltaic component generates clean energy with a levelized cost of electricity (LCOE) of 837 MXN/MWh (47.4 USD/MWh), while diversifying the cropping system enhances resilience against market and climate shocks.

## Graphical abstract

This work presents a rural development strategy that transforms rainfed maize farms into crop‐rotation agrivoltaics systems irrigated with treated wastewater. Results show that electricity sales to the grid improve land‐use efficiency and provide clean energy to local communities. High value‐added crops enhance agricultural resilience, although increased irrigation demand in a large adoption scenario may jeopardize water security.

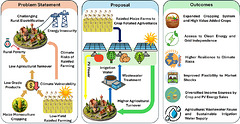

## 1 Introduction

Rural communities represent a critical bottleneck to sustainable developmet, as the majority of the world's poor live in these areas [1]. Despite the essential role of food production by family‐owned farmers [2], over 83% of the poor population reside in rural areas [3]. Low population density and distance from urban zones can damage rural livelihood and cause social decline [4]. The combination of poverty and infrastructure development challenges for distributed settlements resulted in an entanglement in many countries worldwide. In 2022, developing countries spent approximately 10% of their GDP (USD 842 billion) supplying subsidized fuel‐based energy carriers to these communities, trade‐offs that have consistently undermined the true potential of rural communities [5].

Mexico represents a unique profile in the rural context among peer countries. Despite having 21 million hectares of agricultural land and access to the extensive USA marketplace, the agroindustry shows imbalanced development that varies significantly across states [6]. Recent analysis of Mexican agriculture reveals the critical importance of underserved communities, where the politically accessible USA market remains geographically inaccessible [7]. Thus, instead of diversifying production, local farmers cultivate staple crops, primarily rainfed maize, to fulfill local food demand, constrained by poverty‐limited purchasing power. Over two‐thirds of the country's maize farming groups produce grain exclusively for self‐consumption using landrace varieties, with only 10% graded as commercial grain [8]. This flawed business model leaves communities vulnerable to climate change, as maize cultivation under rainfed conditions carries substantial risk, thereby worsening rural poverty [6]. Federal government modernization programs aimed at breaking the cycle of inefficient farming have proven largely ineffective. Resources allocated based on farm size, combined with fragmented large acreages resulting from the “Ejido” landownership model (communal land), have led to ineffective funding allocation [9]. Support received has been insufficient for acquiring machinery and has instead been diverted to debt repayment and unnecessary expenses [10].

The energy sector faces parallel challenges. By 2018, Mexico's political landscape redirected the energy sector agenda toward fossil fuels and coal‐based power generation [11]. The government aimed to strengthen the Federal Electricity Commission's (CFE, by its acronym in Spanish) market dominance [12] and initiated interventions with the private sector, including acquiring large enterprises such as Iberdrola, to enhance state influence [13]. These reforms in Mexican power discourse have increased power grid development costs. Currently, aging power generation and transmission infrastructure, coupled with increasing demand, has resulted in scheduled blackouts across many central Mexican regions. In semi‐urban and rural areas, particularly, power systems overhauls are costly and slow, further complicated by unauthorized energy extensions that drain the grid and cause voltage losses [14].

The benefits of integrating renewable energy technologies into the Mexican energy mix have been studied extensively. Beyond significant improvements in electricity's environmental footprint, solar energy's role in alleviating rural poverty in central Mexico has been studied for the first time [15]. Political interventions and government uncertainties are considered key elements in energy transitions [15, 16]. Previous research recommends tailoring renewable energy adoption strategies according to the local energy mix characteristics and implementing decentralized execution to minimize political friction [16]. Considering the emerging challenges in the energy sector alongside persistent rural poverty and agricultural inefficiencies, deploying agrivoltaics (AV) systems among smallholder farmers cultivating rainfed maize offers a comprehensive, decentralized solution addressing both energy insecurity and underdevelopment.

Successful introductory implementations worldwide have made AV systems a rising renewable energy technology (RET), particularly in rural areas [17, 18, 19, 20]. For the Mexican context, AV‐based irrigation for agricultural regions has also been studied [21]. The results provide an insightful analysis of costs and benefits for using AV in irrigation water supply; however, the benefits of this technology for rural improvement as an income source remain unexplored. Additionally, uncertainties in Mexico's renewable energy sector, especially solar power, have increased technology costs [22]. Therefore, a deeper uncertainty analysis is required to fully evaluate this novel approach's viability in a rural context against systemic uncertainties. Through this research, we explore the feasibility of symbiotic agrivoltaics and crop rotation applications in empowering rural Mexico. To this end, rainfed maize farming transitions to high‐value crops with irrigation and an AV system. Water is presumed to be supplied from available treated wastewater rather than being discharged to the environment. The methodology evaluates the Photovoltaic component of AV individually with a baseline scenario and uncertainty analysis. The revenue mechanism accounts for local energy dynamics and price fluctuations. Integrated system performance was analyzed using sustainability indicators.

## 2 Materials and Methods

### 2.1 Study Location

**FIGURE 1:**
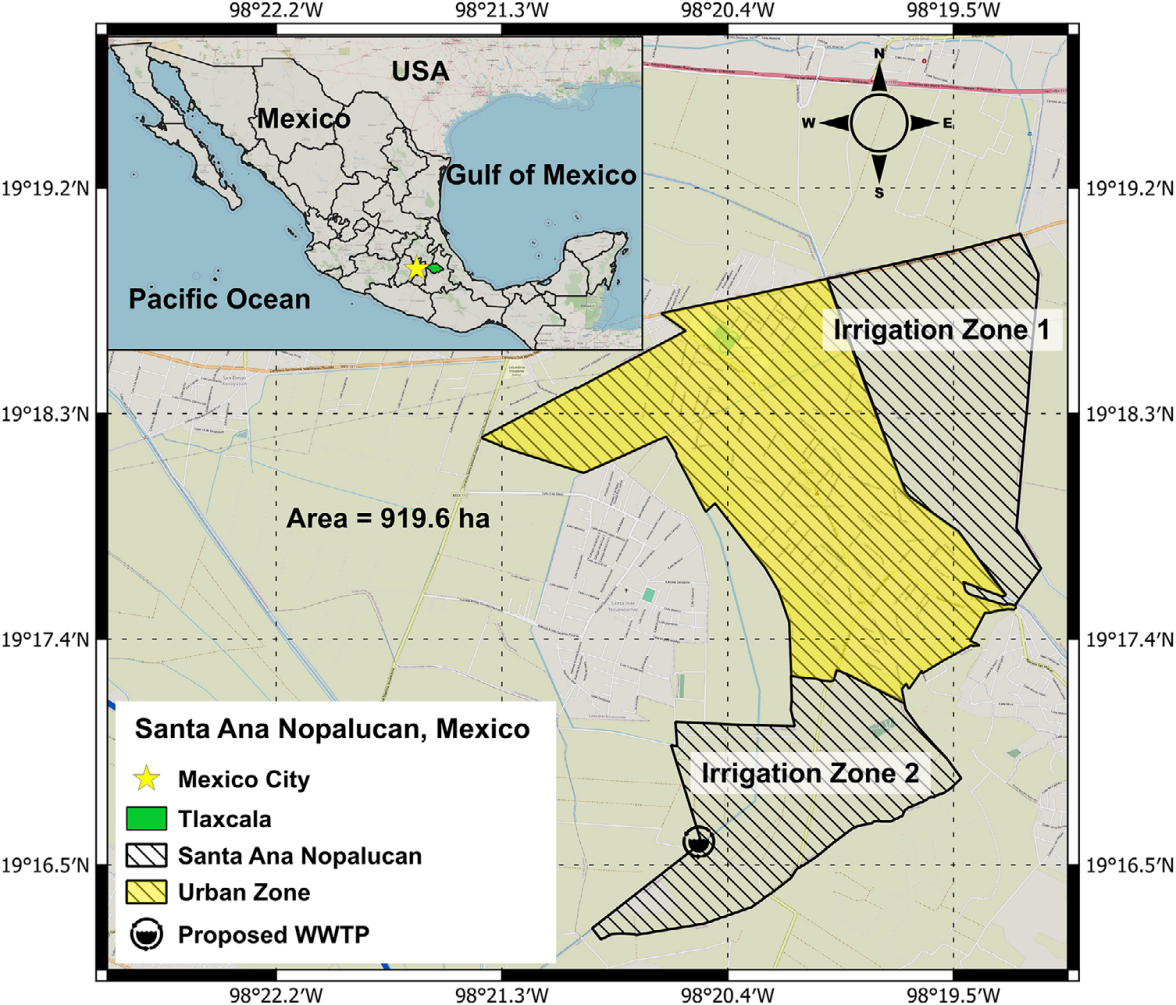
Geographical location of Santa Ana Nopalucan municipality in the state of Tlaxcala and the proposed wastewater treatment plant (WWTP) location.

The selection criteria for evaluating the strategy focused on identifying a single settlement municipality with adequate population size and sufficient agricultural acreage in a less developed state, providing a pragmatic and precise showcase with sufficient resources. Based on these criteria, the municipality of Santa Ana Nopalucan (SAN) (19.30°N–98.33°W) in Tlaxcala state was selected as the case study (Figure 1). SAN is located east of Mexico City, the capital, and had a 2020 population of 7952 residents [23]. To estimate population growth for the project duration, we used the United Nations population growth forecasts for Mexico [24]. The population in SAN is anticipated to increase from 8129 to 9305 residents during the 2023–2047 period. The economic activities portfolio is mainly related to agricultural and related industries, such as machinery and food businesses [25]. Detailed socio‐economic and employment data at the municipal level are unavailable; however, state‐level indicators show Tlaxcala is behind national averages. In 2022, Tlaxcala's Human Development Index (HDI) was 0.772 (ranked 20 of 32), with sub‐indices for education, income, and health at 0.719 (19 out of 32), 0.748 (23 out of 32), and 0.855, respectively [26].

### 2.2 Water‐Energy‐Food System Profiles

This section describes the status of water, energy, and food systems, corresponding supply and demand profiles for 2023, and the basis for projections in the study timeline.

#### 2.2.1 Water System

The municipality is within the Atoyac aquifer system, which has an estimated annual recharge of 212.4 hm^3^ and natural discharge commitment of 41.0 hm^3^, resulting in a surplus of 171.4 hm^3^ for anthropogenic uses. Considering the population in the aquifer zone (2 988 987 residents), per capita annual groundwater availability is estimated at 57.3 m^3^, while 48.4 m^3^ is abstracted through the water rights granted by governmental entities [27]. The annual average precipitation and evaporation rates for the hydrological system are 878.3 and 1150 mm, respectively [27]. According to Meteonorm V8.0, precipitation in SAN is higher, with 1056 mm, with approximately 75.9% of rainfall occurring between June and September. Water demand is estimated using global averages for rural communities; a 75 L/day·capita (average value of 50 and 100 L/day·capita) is assumed for the consumption [28]. Accordingly, for domestic wastewater generation, a conversion rate of 80% is applied to the consumed water [29]. While SAN lacks a dedicated WWTP due to the centralization strategies of wastewater management in Tlaxcala [30], recent discussions have proposed installing a dedicated unit at the confluence of the wastewater channels of SAN and Santa Inés Tecuexcomac, for safe recovery and agricultural reuse [31]. To account for the potential impact of this future unit on irrigation water supply, the irrigation supply point was set at the confluence zone of the two channels (Figure 1).

#### 2.2.2 Agricultural Portfolio

Agricultural profiling data were obtained from the Agri‐food information and inquiry system (SIACON, by its acronym in Spanish) software tool for the year 2023, the latest available version. The following sub‐sections describe water demand estimation, crop acreage, prices, yields, and the crop rotation system. From 2014 to 2023, the agricultural acreage varied, with an average of 341 hectares during the main period of the spring‐summer harvesting season. In 2023, the total cultivated area was 370 hectares, which included 275 hectares under rainfed conditions and 95 hectares under irrigation. Additionally, 10 hectares were cultivated for a second time during the fall‐winter season using irrigation. Maize farming occupies most of the cultivated land, representing 274 out of the 370 hectares, in which 74.2% is rainfed, and the average yield is 3.07 ton/ha. The detailed profile of the agricultural profile in 2023 for SAN is reported in Table S1.

#### 2.2.3 Energy System

According to energy projects reported in literature [32], the only energy project deployed for the public sector in Tlaxcala is a 200 MW PV unit, installed in Calpulalpan municipality, in the northern zone of the state. Therefore, it is expected that Tlaxcala state will be highly dependent on the national power grid. On the demand side, per capita electricity demand was estimated using the hourly energy forecast (Pronóstico de la Demanda in Spanish) derived from the Day‐Ahead Market allocation process (Asignación Suplementaria de Unidades de Central Eléctrica para Confiabilidad in Spanish) for the state of Tlaxcala for the reference year 2023 (Supporting Data – “per capita energy demand” spreadsheet), for a precise and spatially relevant basis for the demand projections [33]. Based on the state population estimate, the calculated per capita electricity demand was 2522 kWh/capita, which is slightly higher than the reported 2420 kWh/capita [34], likely due to the inclusion of additional load sources at the state level. It is worth noting that, given the relative socio‐economic uniformity across Tlaxcala due to its small geographical size, SAN's per capita electricity demand was assumed to follow the state average. The hourly demand profile was subsequently normalized and projected using forecasts from the Mexican Energy Ministry, which estimates annual per capita electricity demand growth rates of 2.4% under the baseline scenario, and 2.1% and 2.9% under the lower‐ and upper‐bound scenarios, respectively [35].

### 2.3 Strategy Outline and Intervention

**FIGURE 2:**
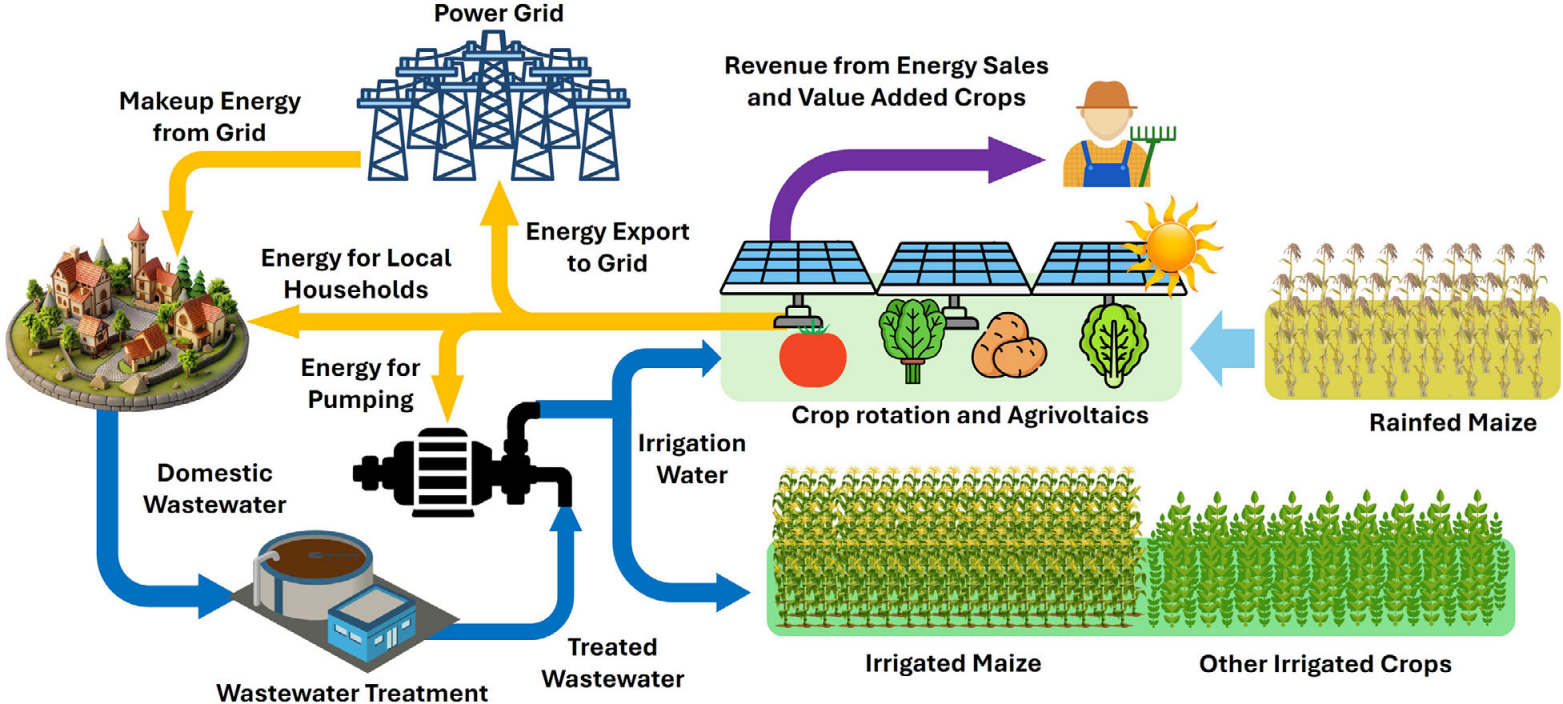
AV‐powered rural electrification and development strategy outline.

Considering supply and demand profiles for the WEF sectors as well as the projected population growth, we developed this strategy to evaluate the costs and benefits of agrivoltaics coupled with a crop rotation system in SAN to partially transform rainfed maize farms. The strategy outline is demonstrated in Figure 2.

#### 2.3.1 Agrivoltaics System Design and Performance Modeling

**FIGURE 3:**
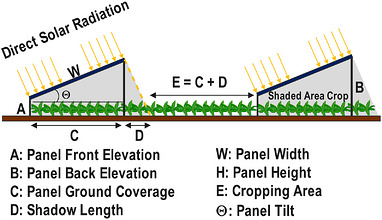
The configuration of a half‐density AV system.

Various AV configurations have been proposed for different cropping systems. While crop tolerance to shading and solar availability beneath the AV canopy are critical design factors, the half‐density configuration, achieved by removing one PV array chain out of every two in a full‐density setup, is often considered the most suitable option. This arrangement has been shown to reduce solar irradiance by up to 30% [36] while maintaining a high Light Productivity Factor (LPF) [37], making it appropriate for light‐sensitive crop species. Several empirical studies have adopted this configuration as their AV design [17, 36, 38, 39]; therefore, the present study employed a horizontal half‐density AV layout (Figure 3) following the design reported by [40, 41]. The area occupied by each panel and the associated area allocated for crop cultivation are calculated using Equations (1) and (2) based on the shading cast from the normal beams reaching the panel surface. The Balance‐of‐System (BOS) structure for the AV system is technically identical to ground‐mounted systems, and half of the area will be shaded, and associated crop losses will be applied to the shaded area.

(1) $$D=\left(A+W\times \sin\theta \right)\;\times \frac{\cos\left(90^{\circ }-\theta \right)}{\cos\theta }$$

(2) $$A_{\mathrm{panel}}=A_{\mathrm{cropping}}=\left(W\times \cos\theta +D\right)\;\times H$$

where *A* is the front elevation of the solar panel and is set at 0.5 m; *W* and *H* are the panel's width and height, equal to 1.134 and 2.274 m, as obtained from the panel's data sheet; θ is the panel's tilt and is equal to 22°, the recommended tilt by PVGIS for the study coordination. Thereby, *D*, the shadow line is calculated to be 0.37 m (Equation 1). Last, *A*_panel_ and *A*_cropping_ are mathematically equivalent due to the half‐density AV array and are equal to 3.241 m^2^. For a 1 ha half‐density AV unit, it is estimated that 1542 solar panels could be installed with an overall direct current (DC) capacity of 848.1 kW, and the remaining 0.5 ha will be devoted to farming. Energy output modeling of the PV array was performed using the PVlib Python library, following an approach similar to that described in ref. [42]. Detailed calculation procedures are provided in Section S2 and Table S2.

#### 2.3.2 Crop Rotation in AV System

For farms where AV systems are proposed, we developed a crop rotation system as an alternative for rainfed maize. Thereby, an *ex‐ante* rotation system was developed based on a) local climate conditions, b) revenue for local farmers, c) former successful application in AV systems, and d) avoiding immediate repetition of crop families to prevent phytopathogen damage. To that end, lettuce (*Lactuca sativa*), potato (*Solanum tuberosum*), spinach (*Spinacia oleracea*), and tomato (*Solanum lycopersicum*) were selected sequentially for the proposed crop rotation system.

#### 2.3.3 Irrigation System Standardization

Given the identified inefficiencies in the agricultural system, specifically in underserved communities [7], we calculated the standard water demand for all current cultivated crops and crops proposed in the crop rotation system. First, the cropping calendar developed for the Atoyac River basin has been utilized for outlining the different growth stages of crops according to local environmental conditions [43]. The FAO's CROPWAT model was then used to estimate crop water requirements [44]. This model uses the Penman–Monteith equation to calculate crop evapotranspiration (ETc). The rainfall pattern was integrated into the model to determine the amount of freshwater needed to meet the ETc. The necessary meteorological data were obtained from Meteonorm V8.0. Lastly, a loss of 20% was attributed to considering the expected unrecoverable losses in the irrigation system's operation, which was incorporated into the freshwater resources required [45]. The energy required for pumping is dynamically calculated by accounting for both the static lift head and the friction losses associated with the distance between each irrigation point and the WWTP. The methodology developed and applied for this section is detailed in Section S3.

#### 2.3.4 Crop Yield Under AV Canopy

The AV system's canopy casts shade on the crops beneath, resulting in a lower photosynthesis rate compared to open‐field conditions. To calculate the crop's yield losses in shading conditions, we applied a modified version of the LPF model developed in [37], by integrating Spitter's relationship to the solar radiation reaching beneath the AV canopy and using the light sharing ratio of crops under the AV canopy. The model uses the ratio of Photosynthetically Active Radiation (PAR) received by the crop's surface with and without the AV system. To that end, the model aggregates the amount of useful PAR received on the crop's surface versus the threshold PAR for the crop. The modification applied to the under‐canopy solar ray calculation, in which we applied Spitter's relationship to estimate the diffuse fraction of the PAR reaching under the canopy [46]. For this calculation, we used the hourly Typical Meteorological Year (TMY) data retrieved from PVGIS for SAN. Then the open‐field PAR vector is calculated using Equation (3).

(3) $$\mathrm{PA}R_{\mathrm{open}}\;\left(t\right)=0.5\times \mathrm{GHI}\left(t\right)$$

where 0.5 represents the approximate proportion of sunlight corresponding to the photosynthetically active spectrum (400–700 nm) [47]; GHI(t) is the global horizontal irradiance vector in (W/m^2^). To estimate the diffuse PAR beneath the AV canopy, the ratio of diffuse horizontal irradiance (DHI(t)) to GHI(t) was used to compute the open field diffuse fraction (DF) vector. Next, the combination of DF and solar zenith angle vectors was then applied to Spitters’ relationship to determine the diffuse radiation fraction transmitted under the canopy. The resulting vector was subsequently applied to PAR_open_(t) to calculate the portion of PAR available for crops beneath the AV system (PAR_AV_(t)). The two PAR vectors were generated and then compiled in Equation (4) according to each crop's cultivation and harvesting timeframe, and the total PAR used by each crop has been calculated.

(4) $$Y_{\mathrm{PAR},i}=\frac{\sum_{C,i}^{H,i}\mathrm{MIN}\left(\mathrm{PA}R_{\mathrm{AV}}\left(t\right),\;\mathrm{PA}R_{\mathrm{th},i}\right)}{\sum_{C,i}^{H,i}\mathrm{MIN}\left(\mathrm{PA}R_{\mathrm{open}}\left(t\right),\;\mathrm{PA}R_{\mathrm{th},i}\right)}$$

where H, and C, i are the harvesting and cultivation dates for crop i, obtained from Table S3; PAR_th,i_ is the threshold PAR value for each crop i, in (W/m^2^), corresponding to 130, 87, 87, and 202 for tomato, potato, spinach, and lettuce, respectively [48]. The calculated Y_PAR,i_ demonstrates how much PAR is reduced between open‐field and beneath the AV canopy. This ratio was used as a proxy to estimate the relative yield performance of crops grown under an AV canopy compared to open‐field crops.

The AV canopy can also reduce irrigation water demand. Previous studies have investigated this effect through experimental analyses and comparisons with control farms [36, 38, 39, 40]. As shown in Table S3, irrigation demand depends strongly on rainfall patterns, and ETc alone does not precisely reflect freshwater requirements. Moreover, to the best of our knowledge, no theoretical modeling framework currently exists that can be applied within the scope of this study to accurately estimate under‐canopy ETc. Therefore, considering the rural electrification and poverty context of this study, we adopted a conservative assumption by using the irrigation water demand values from open‐field conditions for the shaded area of the AV system, acknowledging this as a limitation of the methodology.

#### 2.3.5 Scenario Development

Three different adoption rates have been considered as scenarios to account for farmers’ affordability constraints and willingness to accept new technologies. These scenarios represent high, medium, and low adoption rates of AV by 15%, 10%, and 5% of rainfed maize acreage, respectively, alongside a Business as Usual (BAU) scenario. The land use profile associated with each scenario is available in Table 1.

**TABLE 1:** Land acreage profile according to the scenarios developed.

Crop acreage (ha)	BAU		Scenario A		Scenario B		Scenario C	
	Irrigation	Rainfed	Irrigation	Rainfed	Irrigation	Rainfed	Irrigation	Rainfed
Beans	2	42	2	42	2	42	2	42
Green Barley Forage	10	10	10	10	10	10	10	10
Green Broad Beans	4	7	4	7	4	7	4	7
Maize Grain	75	216	75	205.2	75	194.4	75	183.6
Vetch	4	—	4	—	4	—	4	—
Tomato	—	—	2.7^a^	—	5.4^a^	—	8.1^a^	—
Potato	—	—	2.7^a^	—	5.4^a^	—	8.1^a^	—
Spinach	—	—	2.7^a^	—	5.4^a^	—	8.1^a^	—
Lettuce	—	—	2.7^a^	—	5.4^a^	—	8.1^a^	—
AV	—	—	5.4	—	10.8	—	16.2	—

^a^ 50% of the land is shaded, therefore yields are adjusted according to expected losses for the shaded parts.

### 2.4 Techno‐Economic Analysis

#### 2.4.1 Techno‐Economic and Uncertainty Analysis of AV Systems

The techno‐economic analysis was conducted under both baseline and uncertainty scenarios using the most recent data available for Mexican PV projects from the IRENA report [22]. Two feasibility indicators were employed, first Net Present Value (NPV) as a measure of project profitability, and second, the Levelized Cost of Electricity (LCOE) to assess the cost‐effectiveness of generated power. The revenue structure was defined based on three sources: electricity supplied for water pumping at a fixed tariff of 2.782 MXN/kWh under the agricultural electricity [49]; electricity sales to local households and grid exports using the dynamic hourly pricing of the grid node located near SAN, to which the municipality is connected [50]; and revenues from Clean Energy Certificates (CELs). CEL revenues were incorporated assuming a price of 20.57 USD/CEL and an allocation rate of 1 CEL/MWh, in accordance with the long‐term power purchase agreements issued by the Mexican government in 2017 [51].

Although the applied CEL value corresponds to the most recent publicly available market price, recent regulations establishing a 13.9% decarbonization threshold across Mexican sectors are expected to sustain demand for CELs. For comparison, the regulatory threshold in 2018 was only 5.0%, indicating a substantial increase in future clean energy certificate demand [52]. Importantly, among the three projected growth rates for per capita electricity demand in Mexico discussed in Section 2.2.3, only the baseline scenario growth rate of 2.4% was incorporated into the techno‐economic analysis.

The uncertainty in the techno‐economic factors is evaluated by a sensitivity analysis and a Monte Carlo simulation. Thus, first, a ± 25% applied to the cost, revenue, and financial elements. The Monte Carlo simulation was accordingly applied based on the maximum, minimum, and average values for the cost benchmarks for the OECD countries using the PERT distribution function. For the revenue items, we used a ± 25% deviation and generated random samples for a 10 000‐iteration Monte Carlo simulation. It is important to note that, in addition to the factors considered in the baseline techno‐economic analysis, a curtailment factor was applied to the electricity exported to the grid after fulfilling the pumping and household demands, to account for the technical limitations of the power grid infrastructure. Given the MXN basis of the analysis, a 17.69 MXN/USD rate is applied to the USD values [53]. A detailed description of the calculation procedures, as well as the inputs incorporated in the techno‐economic analysis are represented in Section S4 and Table S4.

#### 2.4.2 Farm Economics

Farm economics were analyzed based on the lifetime aggregated revenue derived from both agriproduct and energy sales. In the previous study, SAN was estimated to have the potential to achieve maize yields exceeding 13.2 ton/ha [43]; however, within the context and timeframe of the present study, this value may represent an overestimation. Therefore, we adopted the state‐average maize yield under conditions of sufficient irrigation supply. Similarly, the yields and market prices of the crops proposed in the rotation system were assumed to follow state‐level averages due to the lack of location‐specific data for SAN. The yield, price, and profit margins used, along with the corresponding references for farm economics, are presented in Table S5. The total farm economics is calculated using the NPV of agriproduct sales and energy export turnover over the project's lifetime using Equation (5).

(5) $$\mathrm{FE}\;=\sum_{t\;=1}^{30}\left[\frac{\sum \left(A_{j}\times Y_{j}\times P_{j}\right)}{{\left(1+r\right)}^{t}}\right]+\mathrm{NP}V_{\mathrm{AV}}$$

where A is the area of each specific crop in hectares (ha); Y is the yield of each crop in (ton/ha), and P is the price of the agricultural product in (MXN/ton); NPV_AV_ is the NPV calculated from revenues generated through energy sales. The sub‐index j indicates the watering regime, irrigation and rainfed and r represents the discount rate, set at 6.5%, and t is the year counter.

For accurate estimation of crop prices over the study period, we analyzed the price trends of agricultural products from 2014 to 2023, considering the inflation rates in both Mexican Peso (MXN) and USD. However, we did not observe any consistent trends in either currency. This forced us to exclude inflation, similar to electricity prices, and adopt the MXN 2023 basis for the crop price analysis. Specifically, we focused on prices for locally cultivated crops in the SAN area and for crops included in the crop rotation plan in national averages.

### 2.5 Sustainability Analysis

#### 2.5.1 Crop Diversification

Diversifying the crop portfolio helps to improve the resilience of agriculture against both climate and market shocks. Adding cash crops like tomato to rainfed maize systems increases overall diversity, which not only reduces vulnerability to extreme weather but also improves income rate for smallholder communities. To assess this, we used two diversity indicators. The first is the Shannon–Wiener Diversity Index (H′), which measures the number of different crops and the presence of less common ones in the system. The second is Simpson's Diversity Index (D), which emphasizes whether a few crops dominate or whether crops are more evenly balanced. The formulas used to calculate H′ and D are shown in Equations (6) and (7), respectively.

(6) $$H^{\prime }=-\sum_{n\;=1}^{N}p_{i}\times \ln p_{i}$$

(7) $$D\;=1-\sum_{n\;=1}^{N}{p_{i}}^{2}$$

where p_i_ is the acreage share of each crop from the entire agricultural acreage and N is the total number of species.

#### 2.5.2 Grid Independence

Measuring self‐sufficiency in the energy system involves evaluating how effectively each scenario can meet the increasing energy demand of households. Energy security is measured based on the share of AV's photovoltaic power within the local energy mix. To this end, an hourly energy balance was calculated by matching the developed load profile, described in Section 2.2.3 and comprising both irrigation and household power demand vs. the PV generation profile. In hours where PV generation falls short of demand, the resulting deficit is covered through grid imports. The proportion of total power demand met by PV generation is therefore defined as the grid independence index, as represented in Equation (8).

(8) $$GI\%\;=\frac{\sum_{t=1}^{8760}\min\left(PV_{t},H_{t}+I_{t}\right)}{\sum_{t=1}^{8760}\left(H_{t}+I_{t}\right)}\times 100$$

where GI% represents the annual grid independence percentage (%); *PV_t_* represents the hourly PV energy profile in each deployment scenario (kWh); *H_t_* represents the household's hourly energy demand (kWh) for SAN households; *I_t_* represents the hourly energy demand for pumping water from WWTP to the farms (kWh); and *t* is the hour counter.

#### 2.5.3 Food Security

Grains are essential for ensuring food security, and in the Mexican context, maize is a fundamental component of national cuisine. To assess the availability of maize for food demand, we compare the total supply of maize from local farming to the population and local food demand. Despite the historical demand of 127 kg/(capita·year) for Mexico [54], a more recent record of 120.5 kg/(capita·year) is used to reflect the changes in food preferences in the past decade [55].

#### 2.5.4 Water Circularity

The only water source considered to meet the irrigation demands of both existing irrigated fields and proposed AV fields is the recovered treated wastewater from the centralized WWTP located in the southern part of the study area. The extent to which treated wastewater can fully or partially meet irrigation demand, the water security index (WSI) is defined as the self‐sufficiency indicator according to the available wastewater to fulfill the irrigation demand (Equation 9). Greater reuse of treated wastewater increases the circularity, thereby reducing the overall water footprint of the proposed strategy.

(9) $$\mathrm{WSI}\%\;=\frac{\min\left(Q_{demand,i},\;\;Q_{wastewater}\right)}{Q_{demand,i}}\times 100$$

where WSI % is the water security index with a maximum value of 100% if the wastewater resources exceed the agricultural demand (%); *Q*_*demand*, *i*_ and  *Q_wastewater_* are the annual volume of the agricultural demand for scenario *i* and available treated wastewater according to projections in 2023 basis (m^3^).

## 3 Results

In this section, the results obtained from the developed methodology are presented and analyzed. Detailed and supporting data are provided in the Supporting Data spreadsheets. The “Hourly Energy Balance” spreadsheet includes the hourly demand profiles for pumping and household electricity consumption, PV generation, and the corresponding grid export and import values. In addition, the “Techno‐Economic Results” spreadsheet contains the detailed results of the techno‐economic analysis for both baseline and uncertainty scenarios, while the “Agrivoltaics Crop Results” spreadsheet presents the PAR reduction estimations and projected yield losses for under‐canopy crops.

### 3.1 Techno‐Economic and Uncertainty Analysis

Table 2 represents the summary of the results obtained from techno‐economic analysis and photovoltaic performance results for the scenarios. Positive NPV values indicate a profitable investment opportunity. Moreover, the projected LCOE is approximately 837 MXN/MWh (47.4 USD/MWh), which is significantly lower than the average LCOE records for other Mexican photovoltaic facilities, recorded at 65 USD/MWh. This record is also comparable to onshore wind units with a cost of 47 USD/MWh [22], since wind systems often benefit from higher capacity factors, which competitively drop the cost of generated electricity.

**TABLE 2:** The results obtained from the techno‐economic analysis of scenarios.

Unit	Power capacity (kW)	CAPEX (MXN)	NPV (MXN)	LCOE (MXN/MWh)	Lifetime CF	Lifetime energy (GWh)
Scenario A	9163.6	170 208 360	34 185 893	837	21.48%	517.4
Scenario B	18 327.6	340 426 935	68 215 102			1034.8
Scenario C	27 491.2	510 635 294	102 245 659			1552.1

The capacity factor values for the first 15 years of operation align with those of other utility‐scale units that have power purchase agreements (PPAs) with the government for the same duration. In this analysis, the unit located in Calpulalpan, in the northern region of Tlaxcala, is expected to outperform the AV systems proposed for SAN. The AV systems are projected to have a capacity factor of 22.4% during their first 15 years of operation. It is estimated that, on average, 17.2, 34.5, and 51.7 GWh/year can be generated under Scenarios A, B, and C, respectively.

Comparing the energy generation performance of the proposed system with other national projects presents challenges. Due to the absence of an integrated reporting system for energy generation at each facility, available records often contain inconsistencies. For example, the Villanueva plant in the state of Coahuila, the largest PV facility in Mexico, with an installed capacity of 754 MW, has been reported with a CF of 25.7% [56], while the World Bank estimated 34% [57], and the Mexican Projects Hub reported 30.6% [32]. Similar discrepancies are observed in other projects, for example, the Kambul plant in Yucatán was reported to have a CF of 21.0% [32, 57], whereas the nearby La Pimienta plant in Campeche recorded 30.0% [58]. Based on these observations, we consider the data provided by official sources, such as the Mexican Projects Hub and the World Bank, to be more reliable. Also, it is more likely due to the higher DC‐to‐AC design ratio, a conventional approach used to maintain inverters’ AC power output over a wider share of the diurnal cycle.

**FIGURE 4:**
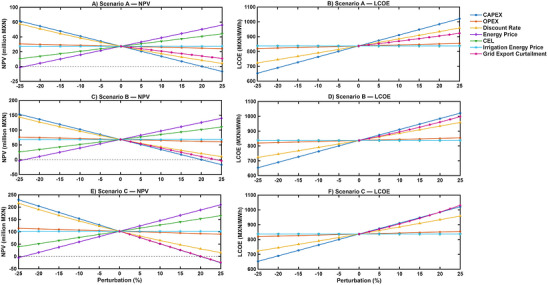
Results of the techno‐economic sensitivity analyses for different AV adoption scenarios, illustrating the response of (A) NPV and (B) LCOE to variations in cost, revenue, and financial parameters.

Figure 4 illustrates the results of the sensitivity analysis for the scenarios. Overall, it is expected that increasing the AV acreage will result in a larger share of electricity being exported to the grid. Consequently, the curtailment factor would lead to greater energy losses, decreasing project revenues, and increasing the cost of delivered electricity. First, the NPV sensitivity analysis indicates that, under the upper‐limit of curtailment rate and CAPEX, all scenarios may become unprofitable. The results also highlight the importance of support mechanisms, such as CAPEX subsidies and low‐cost financing which can substantially improve project profitability. Projections show that a 25% reduction in either the discount rate or CAPEX could approximately double the project's NPV. Importantly, the electricity price of pumping has the lowest impact on NPV's sensitivity. Considering the affordability limitations in underserved communities, the energy costs for pumping could be reduced or partially offset to support farmers without compromising on the project's profitability.

The LCOE sensitivity analysis demonstrates that the projected ranges are relatively comparable across all scenarios. Regardless of the AV adoption rate, the projected LCOE values range between 653 and 1031 MXN/MWh. CAPEX and discount rate remain the most influential parameters in Scenarios A and B; however, in Scenario C, the impact of the curtailment factor surpasses both variables. For both indicators, OPEX demonstrated the second‐lowest impact on the sensitivity analyses results. Table 3 represents the distribution of the results from the Monte Carlo simulation, which indicates a positive NPV ranging from 97.7% in Scenario A to 89.5% in Scenario C. In Scenario A, more than 72.7% of the simulated NPV values surpass the baseline performance, while this ratio decreased to 64.6% and 61.0% for Scenarios B and C, respectively. Lastly, the likelihood of negative NPVs was estimated at 5.2%, 8.5%, and 10.5% for Scenarios A, B, and C, respectively.

**TABLE 3:** The details of the Monte Carlo simulation.

	NPV (million MXN)						LCOE (MXN/MWh)					
Scenario	mean	min	5%	50%	95%	max	mean	min	5%	50%	95%	max
A	56.7	−50.3	−0.5	55.8	117.7	176.9	747	490	596	740	923	1088
B	97.9	−136.5	−17.0	95.5	221.2	360.5	773	511	612	764	966	1219
C	136.2	−221.9	−38.4	133.0	325.1	518.7	787	488	616	778	990	1264

For LCOE, the behavior observed in the Monte Carlo and sensitivity analyses was similar. In the baseline scenario, the curtailment rate was assumed to be zero, which resulted in identical LCOE values (837 MXN/MWh) across all scenarios. Although this assumption may overlook the local grid's technical limitations, the comparison indicates that 68.7% of the simulated LCOE values, even under the worst‐case scenario (Scenario C), remain lower than the baseline estimate. Overall, the 5th to 95th percentile range of the Monte Carlo simulation results spans from 608 to 959 MXN/MWh, with an average LCOE of 769 MXN/MWh.

### 3.2 Grid Independence

**FIGURE 5:**
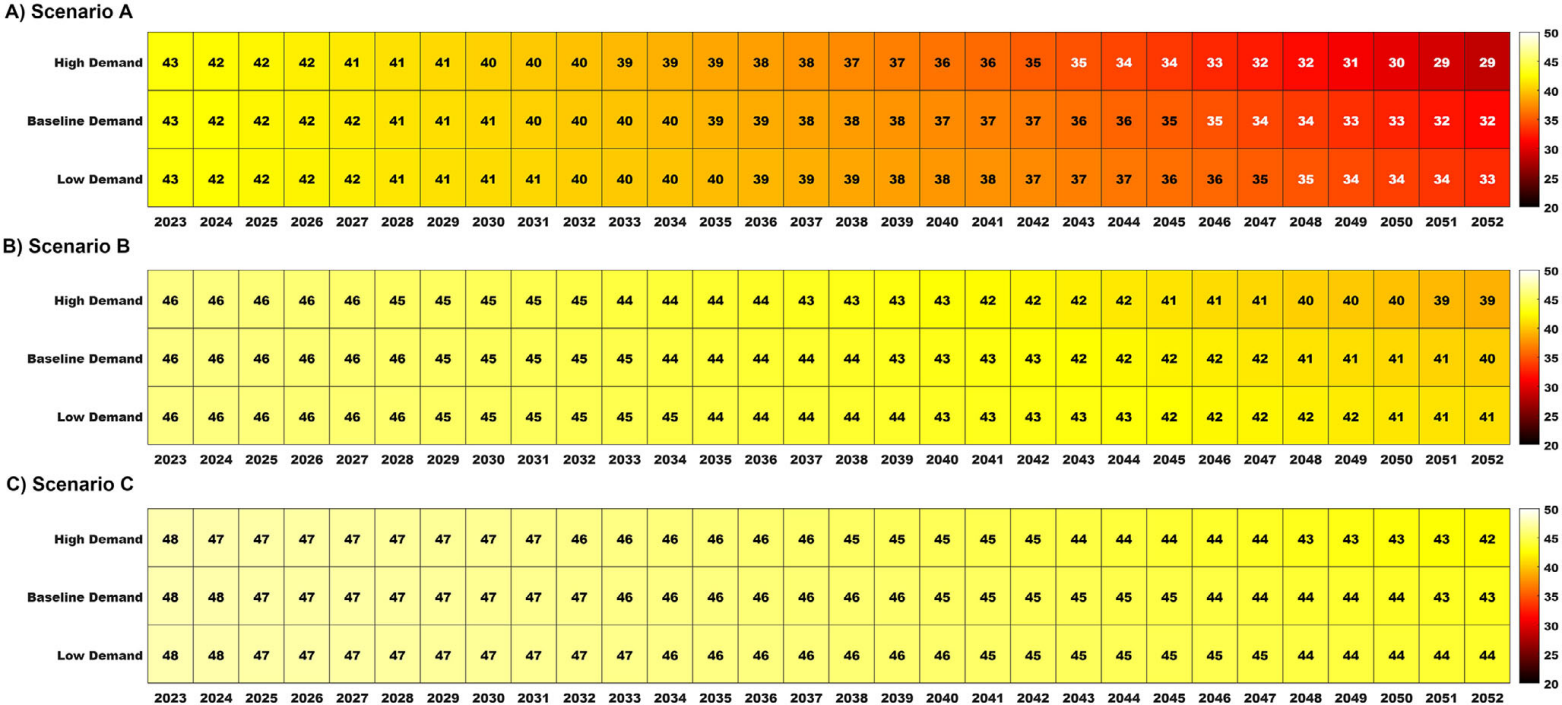
Grid Independence projections according to the different adoption and demand scenarios. (A) Scenario A‐5%; (B) Scenario B‐10%; (C) Scenario C‐15%.

The grid independence projections are demonstrated in Figure 5. Accordingly, all adoption scenarios could supply approximately 40% of the local electricity demand for more than 15 years. Meanwhile, Scenario C demonstrates a more saturated level of grid independence throughout the project's lifetime, highlighting a critical aspect of the local energy consumption pattern. Approximately 45% of the total electricity demand in SAN (more broadly, Tlaxcala) occurs during daylight hours. It can be concluded that nearly half of the electricity demand could be directly supplied by PV generation without requiring intermediate energy storage systems.

Under the worst‐case combination of high energy demand growth and low AV adoption, a 9.2 MW AV system would still be capable of supplying the entire pumping energy demand and at least 30% of household electricity demand over a 30‐year operational period. In all scenarios, the AV arrays generated sufficient electricity to fully operate the pumping and irrigation systems. The surplus electricity generated by the PV system was exported to the grid to generate additional revenue through exchange with the national power network. Overall, under the baseline electricity demand growth, Scenarios A, B, and C are projected to export a cumulative total of 154.4, 612.8, and 1,108.4 GWh of clean electricity to the grid over the project lifetime, respectively.

### 3.3 Crop Yield in the AV System

**FIGURE 6:**
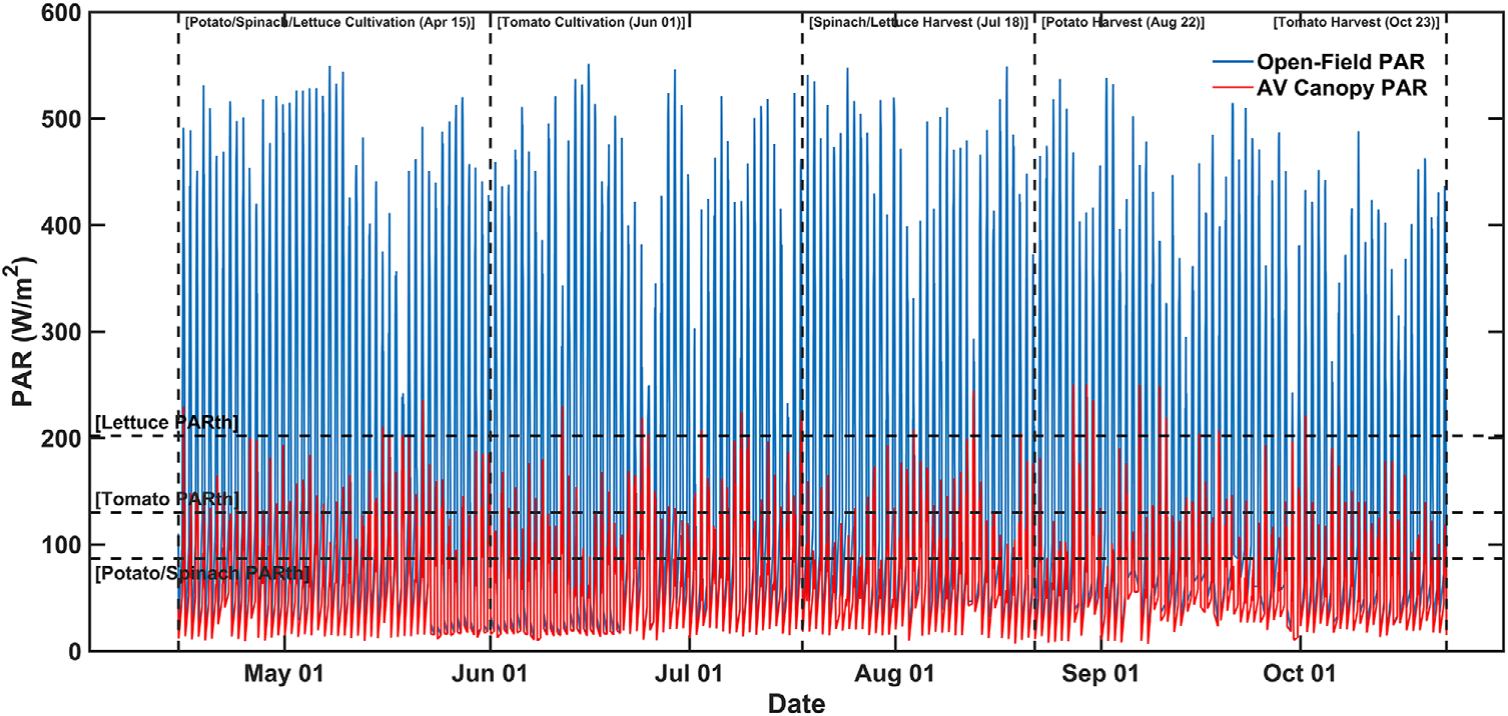
Hourly profile of PAR for open‐field and AV system conditions. Horizontal lines indicate the threshold PAR values for different crops, while vertical lines represent their respective cultivation and harvesting dates.

Figure 6 illustrates the hourly profile of PAR under open‐field and AV canopy conditions for SAN. The analysis indicates that the AV canopy can reduce PAR by up to 90.8% during certain hours of the day. Across the entire cropping season (April 15–October 23), the total PAR flux for the open‐field condition is estimated at 578.2 kWh/m^2^, which decreases to 199.2 kWh/m^2^ under the AV canopy, representing an overall reduction of 65.5%. However, the actual yield reduction depends on crop‐specific PAR thresholds and the temporal distribution of available light.

Given the high PAR threshold values of tomato and lettuce, their yields are expected to decrease by approximately 44.8% and 26.5%, respectively. Elamri et al., (2018) reported similar reductions (21.6%–30.8%) for comparable AV configurations in France [40]. This agreement can be explained by the PAR threshold range for lettuce (186–202 W/m^2^); in this study, we used the upper limit (202 W/m^2^) to avoid overestimating farm revenues. When the lower threshold (186 W/m^2^) is applied, the yield reduction for lettuce reaches 41.4%, demonstrating acceptable agreement with empirical findings.

Tomato yield reductions have also been widely studied. Dal Prà et al., (2025) found that for a ground coverage ratio (GCR) of 41%, similar to our configuration, tomato yields declined by 24.5% in Italy [38], consistent with the present analysis based on Spitters’ relationship. For potato and spinach, the yield reduction estimations are 11.9% and 11.7%, respectively. Weselek et al. (2021) examined AV potato cultivation over two consecutive years and found contrasting outcomes, yields were 20% lower than open‐field conditions during a cooler season due to reduced soil temperature under the canopy, but 11% higher in a subsequent warmer season [36]. The same study reported a 30% PAR reduction under the canopy, which is reasonable given its higher latitude (47.85°N) compared to SAN (19.30°N).

The only conflicting result concerns spinach. Our model estimates an 11.7% yield reduction, while an empirical study in Malaysia reported up to 90% loss under an AV canopy [39]. Since spinach is generally considered a shade‐tolerant crop, such a large reduction could stem from several factors. First, the study sampled spinach over a 5‐week period (“35 days after transplanting”). Although maintaining similar analytical conditions for shaded and non‐shaded crops was necessary, under‐canopy crops typically exhibit delayed maturity under shaded conditions. Therefore, the harvesting conditions should be compared over a longer period to consider the slower growth rate of shaded crops. Second, the Malaysian site (2.22°N) experiences more vertical solar positions (lower zenith angles), which justifies a near‐horizontal panel tilt (10°) and could result in darker shading beneath the canopy. It is important to note that the dense shading conditions led the authors to apply a 5‐week harvesting period.

Overall, we acknowledge the importance of more detailed analyses, particularly regarding the potential limitations of the developed methodology against certain panel tilting configurations. Nevertheless, we adopted the model's result of 11.7% yield reduction for spinach, consistent with the broader literature identifying spinach as a suitable crop for AV systems [59]. Table 4 summarizes the PAR energy flux received by each crop during its respective cultivation and harvesting period, the yield reduction values integrated with the values reported in Table S5, for the share of lands covered by solar panels.

**TABLE 4:** Total PAR values under open‐field and AV system conditions, and the associated yield losses due to shading.

Crop	Cultivation period (Days)	Total PAR_open_ (kWh/m^2^)	Total PAR_AV_ (kWh/m^2^)	*Y_PAR_*	Loss (%)
Tomato	145	191.5	140.7	0.73	26.5
Potato	130	125.5	110.6	0.88	11.9
Spinach	95	92.1	81.3	0.88	11.7
Lettuce	95	187.2	103.4	0.55	44.8

### 3.4 Farm Economics and Crop Diversity

**FIGURE 7:**
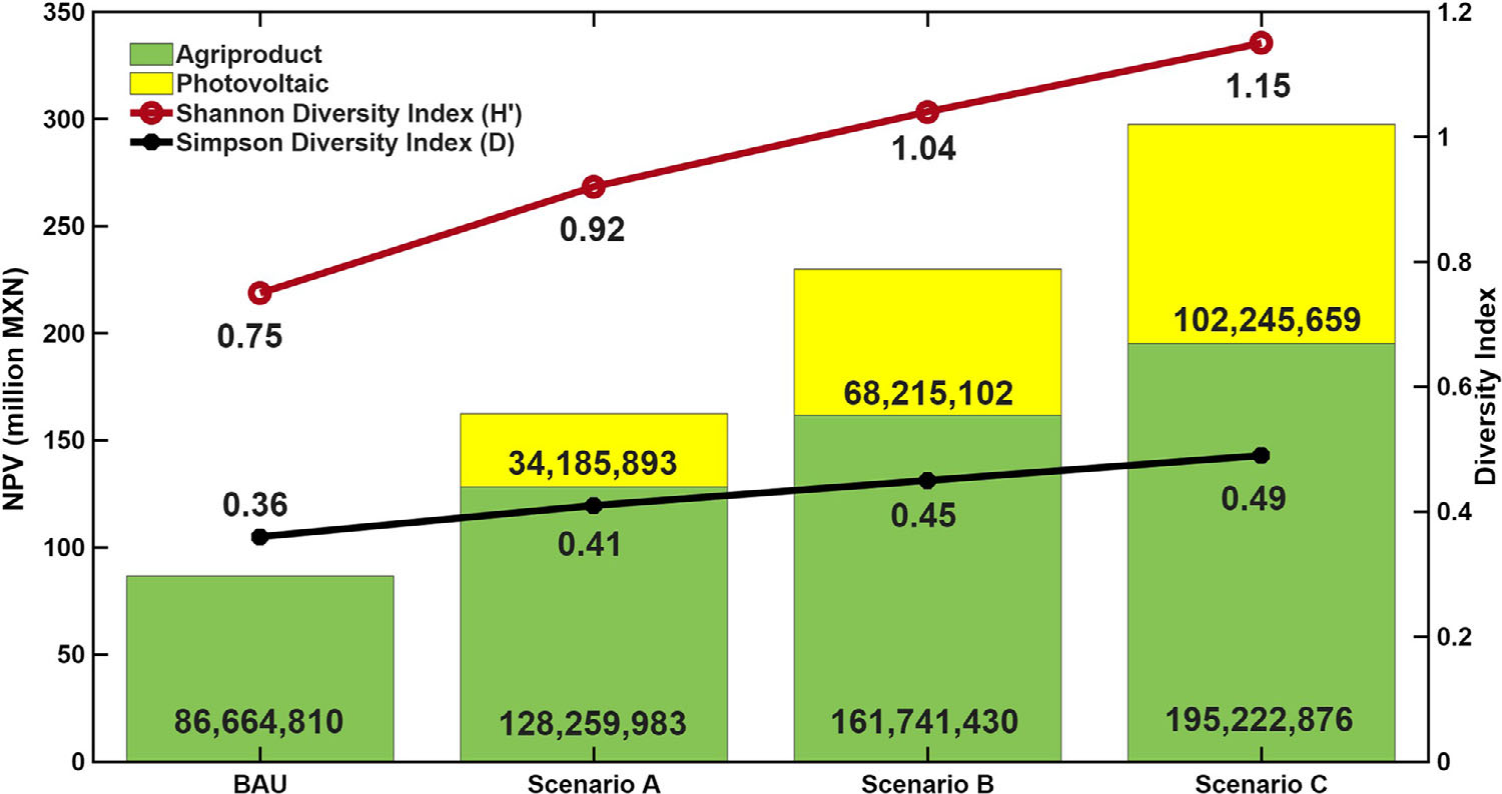
Farm economics and diversity indicators for different adoption and BAU scenarios.

Figure 7 demonstrates farm economics and crop diversity indices for different studied scenarios. An AV system with cash crop achieves higher turnover than rainfed maize, driven by higher yields and market prices. By integrating vegetables such as spinach, lettuce, tomato, and potato, the NPV of agriproduct sales could rise from 86.7 million MXN to 128.2, 161.7, and 195.2 million MXN under Scenarios A, B, and C, respectively. In addition, energy sales could further contribute 34.2, 68.2, and 102.2 million MXN to the NPV across the same scenarios. It is important to note that one hectare of land cultivated solely with rainfed maize can generate an average annual revenue of 17 349 MXN. Contrastingly, the same field can generate an annual gross turnover of 240 260 MXN from agriproduct sales alone. The 30‐year NPV analysis shows that while a rainfed maize farm achieves an NPV of 241 286 MXN, one hectare AV unit can reach as much as 6 506 780 MXN, representing approximately 27 times higher profitability. Therefore, AV systems present unprecedented opportunities for sustainable economic development in rural communities.

In the BAU scenario, Shannon (H′ = 0.75) and Simpson (D = 0.36) diversity both indicate low diversity, with the clear dominance of maize and limited species richness. The main crop, maize, remains highly vulnerable to climate variability, water stress, and market fluctuations [7, 43]. Despite its limited profitability, it continues to be cultivated largely due to cultural significance in Mexican cuisine and the traditionally built local market networks. When new crops are introduced into the portfolio, diversity improves. In Scenario C, H′ rises to 1.15 and D to 0.49, showing a more balanced diversity in the crop varieties and associated acreages. The sharper increase in H′ compared to D highlights the sensitivity of Shannon's index to both richness and evenness, whereas Simpson's index responds more moderately because maize still occupies the majority of the area (258.6 ha compared to 4.05 ha for tomato in Scenario C). This shows that diversification under AV can strengthen resilience, but that dominance of maize farming remains a key weakness of the system.

### 3.5 Food Security

There is a strong domestic demand for maize grain in Mexico, particularly white maize, resulting in one of the highest per capita consumption levels worldwide [60]. Our projections indicate that if Mexican households maintain a consumption of 120 kg per capita per year, maize demand in SAN will rise from 980 tons in 2023 to 1132 tons over the next 30 years. Expanding irrigation on maize farms (75 hectares) and increasing yields to match the state average could boost production. Under the BAU scenario, SAN produces 850 tons annually, leaving a deficit of 130 tons to meet local demand, with improved irrigation, production could rise to 919, 889, and 859 tons per year under Scenarios A, B, and C, respectively.

It is important to note that the negative maize balance, particularly for white maize used for household consumption, must be analyzed from a broader perspective. The vulnerability of Mexican agriculture and food security under maize monoculture and trade liberalization through the North American Free Trade Agreement (NAFTA) has been studied recently [7]. According to these analyses, the challenges associated with maize production and food security represent a national‐level concern. While maize trade with the USA has adversely affected farmers in the central and southern regions by reinforcing a maize lock‐in, farmers in the north diversified their cropping systems, reduced maize cultivation acreage, and benefited from access to the USA market [7].

A large number of Mexican maize producers are small‐scale producers (83% of growers have less than 5 ha) [61, 62] and mainly apply rainfed cultivation to meet their food needs. With increasing precipitation uncertainty and temporal variability in rainfall patterns, these groups are expected to face greater yield losses associated with water stress [6]. Another study conducted for the Atoyac River basin, where SAN is located, estimated through modeling that the region could achieve very high maize yields (approximately 12 ton/ha) due to its favorable climatic conditions for maize cultivation [43]; however, irrigation standardization, reliable water supply, and technological upgrades are necessary to address climatic challenges and, consequently, achieve higher yields. All in all, the deficit balance of maize grain at the municipality level stems from national‐level challenges, and gradual technological upgrades coupled with improved water supply should be considered key priorities for enhancing maize productivity.

It is worth noting that, beyond maize grain production, maize residues (stover) are also a valuable byproduct, particularly for bioethanol and biogas production. In the Tlaxcala region, however, stover is primarily used as cattle feed. Previous simulations estimated that an irrigated maize farm in SAN could theoretically produce 13.2 ton/ha of grain from a total biomass yield of 27.4 ton/ha, resulting in approximately 14.2 ton/ha of stover [43]. Although the stover‐to‐grain ratio in rainfed systems is unknown here, estimations suggest that under the largest maize acreage reduction in Scenario C, stover production could decline by approximately 460 tons. While this reduction is unlikely to disrupt potential industrial activities, it could significantly affect feedstock availability for local dairy and meat producers, potentially increasing dependence on imported yellow maize and associated costs.

### 3.6 Water Circularity

**FIGURE 8:**
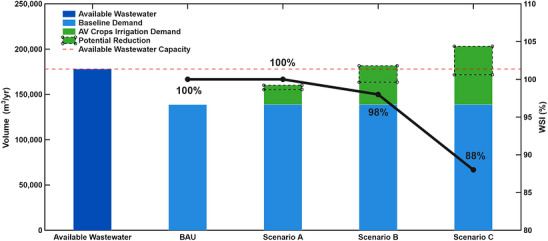
The estimated WSI values according to the projected treated wastewater availability and irrigation water demand under different scenarios in 2023.

The projected Water Security Index (WSI) as well as the water demand volumes for different scenarios and BAU conditions, are illustrated in Figure 8. Additionally, the minimum reported 30% increase in water use efficiency for moderately shaded AV systems (30%–50% shading) was applied solely for demonstrative purposes to illustrate the potential irrigation savings in AV systems [63]. However, this reduction was not incorporated into the water balance calculations. Accordingly, the annual water requirement for existing irrigated acreage is 138 755 m^3^. Each hectare of AV with crop rotation adds 1987 m^3^/year to irrigation demand, as rainfed farming does not rely on freshwater inputs. Consequently, total irrigation demand would rise to 160 217, 181 678, and 203 140 m^3^/year under Scenarios A, B, and C, respectively. On the supply side, the estimated 2023 population of SAN was 8129 residents, and the daily water consumption and sewage conversion rate could generate 178 027 m^3^/year in 2023, with a steady increase to 205 757 m^3^/year in 2052.

Interventions of climate change with higher irrigation demand and changes in domestic water consumption could meaningfully undermine this analysis; however, the observations indicate that a medium‐populated municipality could seek a high‐rate wastewater recovery and agricultural reuse, especially by the implementation of auxiliary elements such as irrigation ponds [43]. Finally, based on the WSI analysis, Scenario A could achieve complete self‐sufficiency with a WSI value of 100%, while Scenarios B and C could reach 98% and 88%, respectively. It is important to note that, assuming an average 23% reduction in irrigation water demand associated with a 30% increase in water use efficiency [63], all scenarios could potentially achieve a freshwater footprint‐free condition through treated wastewater recovery and agricultural reuse, despite uncertainties related to the temporal dynamics of irrigation ETc and under‐canopy microclimatic conditions.

## 4 Discussion

The discussion on AV‐powered rural development can be approached from different perspectives, including renewable energy technology in Mexican energy discourse, considering AVs as a tool to support rural communities’ resilience, adoption pathways, and the affordability of new technology. From a political perspective, the renewable energy sector in Mexico has faced significant challenges since the change in administration in 2018 [16]. A paradigm shift in national energy policy favored fossil fuel technologies [11, 64], while private companies involved in renewable energy experienced market disruptions due to increased governmental intervention, including enterprise acquisitions and strategic redirection toward fossil‐based generation [13]. These conflicts have raised the cost of PV systems in Mexico. Despite global trends of declining photovoltaic costs, there is limited evidence of substantial private‐sector participation in large‐scale PV projects in the country after 2017 [32]. Altogether, despite governmental focus on non‐renewable technologies in the energy sector, under current conditions, the merits of the AV system can be explored from a rural poverty lens.

Over the past decades, the rural empowerment programs aimed at addressing underdevelopment in rural communities have largely fallen short of their intended goals [9, 10]. Today, the low financial profitability of maize farming has escalated tensions between the agricultural ministry and maize producers, forcing authorities to guarantee crop purchases at prices higher than global averages in order to offset production costs [65]. Given these challenges, the photovoltaic component of the AV system alone can substantially improve the economic profitability of rainfed maize farms by diversifying income sources. However, affordability remains a critical barrier, as farmers may be risk‐averse toward adopting unfamiliar technologies. The adoption behavior of farming communities depends strongly on demonstrated prototypes and effective risk management [66]. In this regard, local authorities can play a pivotal role by implementing pilot projects, providing training, and showcasing that the technology is both globally established and locally accessible. Bureaucratic support, particularly in communicating with under‐literate populations, would further help to lower barriers to adoption. Once early successes are demonstrated, word‐of‐mouth can accelerate acceptance within communities, paving the way for larger‐scale deployments. The second expected challenge is financing. Sensitivity analysis revealed that a discount rate of 4.9% could increase the NPV by more than 111%. This suggests that targeted financial support packages for small‐scale deployments could catalyze systematic adoption. Other developing countries have successfully provided low‐cost financial support (e.g., Chile 5.4% and Peru 4%), and even the Villanueva PV project received a support of 4.6%, however, its large‐scale implementation should be acknowledged [57].

For Mexico, an additional opportunity lies in adopting circular economy practices in supplying solar panels. Several utility‐scale PV plants in the northern states are approaching decommissioning, which will generate a flow of dismantled but potentially reusable solar panels. Without proper management, these panels are likely to end up in landfills. However, by implementing quality control protocols and establishing refurbishment facilities, decommissioned panels could be repurposed for rural electrification initiatives such as AV systems. Although this approach presents certain technical and logistical challenges, effective management could significantly enhance the rural electrification and empowerment process.

From an environmental perspective, widespread and decentralized AV deployment is also expected to present challenges related to the management of decommissioned inverters and panels. Specifically for inverters, given their average lifespan of ten years, three inverter waste streams will be generated from each AV unit. The management of e‐waste from PV facilities is an emerging global concern. In Europe, Extended Producer Responsibility (EPR) regulations require manufacturers and distributors to collect and properly manage decommissioned equipment [67]. Similarly, Mexico has established a regulatory framework for e‐waste management under the General Law for the Prevention and Integral Management of Waste (LGPGIR), which assigns shared extended responsibility to producers and distributors of electronic products [68]. However, the implementation of this framework represents inefficient enforcement [69]. Therefore, effective e‐waste management strategies will be essential to ensure that waste generated from non‐industrial‐scale AV systems is safely disposed of or recycled through certified third parties.

## 5 Conclusion

Synergetic energy and food production exhibit a promising pathway to empower underserved rural communities in Mexico. This study examined the potential benefits and challenges of deploying AV systems combined with crop rotation to enhance the economic profitability of rainfed maize farming. The proposal was evaluated through both techno‐economic and sustainability assessments to ensure its long‐term viability. Results indicate that the photovoltaic component of the AV system alone can recover the initial investment and remain profitable over its lifetime. Additionally, the selected crops for the rotation system showed substantial productivity and revenue improvements compared to conventional rainfed maize farming. Additionally, although expanded irrigation will increase the agricultural sector's water footprint, the reuse of locally generated domestic wastewater, after stringent treatment, can offset this demand and strengthen water circularity. Overall, AV systems combined with crop rotation have the potential to significantly improve rural livelihoods, increasing agroindustrial economic turnover by up to 243%. Nevertheless, financial barriers and affordability constraints remain key challenges for underserved communities. Given the government's ongoing approach to supporting rural communities through financial allowances and packages, redirecting these resources toward financing mechanisms that promote self‐sufficiency and long‐term profitability would provide a more effective and sustainable pathway for rural development.

## 6 Limitations and Perspectives for Future Research

The developed methodology may present certain limitations. First, the integration of intermediate technologies, particularly centralized battery energy storage systems under public–community partnership schemes, could further enhance the technical flexibility and operational management of large‐scale implementations in rural communities by more effectively handling surplus electricity generation. Second, as AV systems remain an emerging technology in rural agricultural contexts, farmers’ willingness to adopt such systems should be further investigated through technology diffusion and socio‐economic acceptance studies, particularly considering the potential improvements in agricultural turnover and rural income. Third, large‐scale AV deployment scenarios would require dedicated policymaking regarding photovoltaic power management and pricing mechanisms, particularly through strategies such as Contracts for Difference (CfD), similar to industrial PPAs, and Feed‐in Tariffs (FiT). Lastly, the crop yield loss estimation model, based on proportional PAR reduction, has known limitations for non‐standard installation configurations such as horizontal or vertical panel orientation. More detailed analysis would improve yield predictions under AV canopies, particularly given the relatively lower complexity of such approaches.

## Funding

This work was supported by the Secretaría de Ciencia, Humanidades, Tecnología e Innovación (SECIHTI) through contract No. 2022‐000002‐01NACF‐08059 for the doctoral scholarship of Shahin Rasooli, and through grant project No. PEE 2025 G 203 titled “Maíz sostenible: Sistemas Agrovoltaicos modulares y expansión de irrigación para impulsar rendimientos agrícolas en comunidades rurales de Puebla y Tlaxcala.”

## Conflicts of Interest

The authors declare no conflicts of interest.

## Supporting information




**Supporting File 1**: gch270133‐sup‐0001‐SuppMat.docx.

**Supporting file 2**: gch270133‐sup‐0002‐Data.zip.

## Data Availability

The data that support the findings of this study are available from the corresponding author upon reasonable request.
