# Supplementary material for: Agrivoltaics for Rural Development: Energy Security, Crop Diversification, and Sustainability on Mexican Rainfed Maize Farms
Source: Glob Chall. 2026 Jul 23;10(7):e70133. doi: 10.1002/gch2.70133 (PMC13393070; doi:10.1002/gch2.70133)
Supplement: Supplementary file 1 — Supporting File 1: gch270133‐sup‐0001‐SuppMat.docx. [file GCH2-10-e70133-s001.docx]

**Agrivoltaics for Rural Development: Energy Security, Crop Diversification, and Sustainability on Mexican Rainfed Maize Farms**

Shahin Rasooli^a^, Shahrzad Farhoodi^a^, Catalina Spataru^b^, Pabel Antonio Cervantes-Avilés^a^, Carlos Alberto Huerta-Aguilar^a*^

1. School of Engineering and Sciences, Tecnológico de Monterrey, 72456, Puebla, Mexico
2. Energy Institute, University College London (UCL), UK

* Corresponding author, email address: [huertaa@tec.mx](mailto:huertaa@tec.mx)

1. **Agricultural Portfolio**

The 2023 agricultural portfolio of Santa Ana Nopalucan (SAN), comprising cultivated area, irrigation regime, crop yields, and prices, is provided in Table S 1.

Table S 1. Details of the agricultural portfolio for SAN in 2023 [1].

| **Crop** | **Irrigation** | | | **Rainfed** | | |
| --- | --- | --- | --- | --- | --- | --- |
|  | **Acreage** | **Yield** | **Price** | **Acreage** | **Yield** | **Price** |
| Green Barley Forage | 10.0 | 18.2 | 750.1 | 10.0 | 11.6 | 757.2 |
| Vetch | 4.0 | 22.2 | 522.5 | - | - | - |
| Beans | 2.0 | 1.1 | 11,660.2 | 42.0 | 1.0 | 11,764.4 |
| Green Broad Beans | 4.0 | 3.9 | 6,210.6 | 7.0 | 3.6 | 7,402.1 |
| Maize Grain | 75.0 | 3.4 | 5,893.1 | 216.0 | 2.8 | 6,285.8 |

1. **Photovoltaic modeling**

PV energy modeling was carried out using the PVlib Python library based on the plane-of-array (POA) data retrieved for a panel tilted at 22° and azimuth of -19° (South 0°- East -90°). For this study, we used PVGIS 5.3, the latest version available at the time of preparation. In Mexico, PVGIS provides ERA5-based irradiance since SARAH-3 coverage is limited in this region and pairs it with ERA5/ERA5-Land meteorological datasets. All series were harmonized to hourly local time for PV performance and temperature modeling. PVGIS datasets are validated by JRC (Joint Research Centre) against BSRN ground stations [2]. Given the limited availability of local radiation data and the established quality of PVGIS, we did not perform additional cross-validation for PV performance modeling. As a critical factor, panel temperature was calculated using Faiman’s equation (Eq. 1).

| $T_{p}= T_{a}+ \frac{E}{u_{0}+u_{1}\times WS}$ | Eq. (1) |
| --- | --- |

Where $T_{p}$ and $T_{a}$ are panel and ambient temperatures (°C), $T_{a}$ is retrieved from PVGIS; E is the global irradiance received on the panel’s surface in (W/m^2^). Here, this value is the aggregated energy of POA’s direct and diffuse components; WS is the wind speed in (m/s); $u_{0}$ and $u_{1}$ are the wind independent and dependent heat loss coefficient factors considering convection, conduction, and radiation heat transfer mechanisms (W/(m^2^‧°C)); due to the lack of empirical data for SAN, we used the default values. Then, the effective irradiance of the beam component was computed and employed in the Martin-Ruiz Incidence Angle Modifier (IAM) model. The resulting IAM vector was applied to the direct component of the POA irradiance to estimate the effective irradiance for energy output modeling. The DC and Alternating Current (AC) were calculated using NREL’s PVWatts model. The required data to run the model were retrieved from the datasheet of a commercially available solar panel in the local market, “ET-M772BH550WW/WB” (**Error! Reference source not found.**). It is worth noting that, for the first year, a degradation rate of 2% and 0.58% annual degradation is applied for the solar panel’s efficiency and power output. The project’s lifetime is set at 30 years, as new panel technologies offer a longer operational period [3]. As a performance indicator, Capacity Factor (CF) is calculated for the AV arrays using Eq (2).

| $CF= \frac{Photovoltaic Energy}{8760\times AV Power Capacity}$ | Eq. (2) |
| --- | --- |

Table S 2. Technical data of the employed solar panel for photovoltaic modelling.

| **Parameter** | **Data** |
| --- | --- |
| Manufacturer / Model | EliTe Plus / ET-M772BH550WW/WB 550W |
| Maximum Power – Pmp | 550 W |
| Efficiency | 21.5% |
| Temperature Power Coefficient – Gamma | - 0.34 %/°C |
| Open Circuit Voltage – Voc | 49.9 V |
| Short Circuit Current – Isc | 14 A |
| Panel Degradation Rate | First year 2% - 0.58 %/year |
| Dimensions | 2274 x 1134 x 35 mm |

1. **Irrigation System Standardization**

Irrigation water demand calculation using the developed cropping calendar is represented in Table S 3.

Table S 3. The cropping calendar, crop evapotranspiration, and irrigation water demand [4].

| **Crop** | **CROPWAT Model** | **Cropping**  **Calendar** | **Harvesting Date** | **ETc**  **[m^3^/ha]** | **Effective Rain [m^3^/ha]** | **Irrigation Water*  [m^3^/ha]** |
| --- | --- | --- | --- | --- | --- | --- |
| Beans | Dry Beans | 15-Apr | 2-Aug | 4899 | 3567 | 1790.0 |
| Green Barley Forage | Barley | 15-Apr | 12-Aug | 5439 | 3903 | 2167.5 |
| Green Broad Beans | Green Beans | 1-Jun | 29-Aug | 3572 | 3591 | 803.75 |
| Maize Grain | Maize | 1-Jun | 3-Oct | 4811 | 5282 | 1375 |
| Vetch | Dry Beans | 15-Apr | 2-Aug | 4899 | 3567 | 1790 |
| Tomato | Tomato | 1-Jun | 23-Oct | 6141 | 5827 | 1358.75 |
| Potato | Potato | 15-Apr | 22-Aug | 6144 | 4232 | 2482.5 |
| Spinach | Small Vegetables | 15-Apr | 18-July | 4769 | 3076 | 2053.75 |
| Lettuce | Small Vegetables | 15-Apr | 18-July | 4769 | 3076 | 2053.75 |
| * Irrigation Water is not in balance between ETc and Effective Rain due to the temporal variation of the crop’s water needs. | | | | | | |

The energy required for pumping irrigation water from the WWTP to the farmlands for each AV scenario was calculated using the Total Dynamic Head (TDH). Based on the estimated daily irrigation demand, a pumping schedule from 9:00 to 17:00 was assumed. The non-urban area was divided into two zones, each served by an independent 10-inch PVC pipeline and pump. In QGIS, a 100 m × 100 m grid was developed using the regional Digital Elevation Model (DEM) to estimate the average conveyance distance and static elevation head between the WWTP and the farmlands. Friction losses were calculated dynamically at each hourly time step using the Hazen-Williams equation, reflecting the non-linear relationship between flow rate and head loss. The TDH was then used to compute the hourly pumping energy demand. The complete mathematical formulation is presented in Eq. (3) through Eq. (6).

| $q_{pipe}= \frac{Q}{2\times3600}$ | Eq. (3) |
| --- | --- |
| $\bar{h}_{f}= \frac{10.67 \times\bar{L}\times{q_{pipe}}^{1.852}}{C^{1.852}\times D^{4.87}}$ | Eq. (4) |
| $TDH= \bar{h}_{stat}+1.3\times\bar{h}_{f}$ | Eq. (5) |
| $P_{hyd}=\frac{9.81\times Q\times TDH}{3600\times\eta}$ | Eq. (6) |

Where $q_{pipe}$ is the volumetric flow rate per pump (m³/s); $\bar{h}_{f}$ is the average friction head loss (m); $\bar{L}$ is the average pipe length from the WWTP to the grid cells, equal to 2,382 m; C is the Hazen-Williams roughness coefficient, taken as 150 for PVC pipes; D is the pipe diameter, equal to 10 inches (0.254 m); TDH is the total dynamic head (m); $\bar{h}_{stat}$ is the elevation difference between the WWTP and the grid cells is equal to 4.51 m; the factor 1.3 represents a 30% overdesign factor to account for uncertainties in the pipeline design; $P_{hyd}$ is the energy required for pumping (kWh); and $\eta$ is the pump efficiency, assumed to be 0.65.

1. **Techno-Economic Analysis**

Cost data for techno-economic analysis were obtained from IRENA’s latest benchmark published for Mexico for 2023 [5]. Revenue calculation is based on two components: the exported power to the grid on an hourly basis for the local grid serving the nearby communities, based on dynamic hourly wholesale electricity market prices for the node 02LOA-115 [6] (Supplementary Data, “PML prices” spreadsheet); and clean energy certificates (CELs) allocated based on generated clean energy with pricing of 20.57 USD/CEL and a rate of 1 CEL/MWh. It is worth noting that electricity pricing behavior over the past six years reflects non-economic interventions, including governmental suppressions on pricing [7]. Therefore, energy prices in this study were aligned with the 2023 cost baseline. For operation and maintenance expenses (OPEX), we used the global median value of 7.56 USD/(kW‧year).

We evaluated the techno-economic performance of the proposal using Net Present Value (NPV) and Levelized Cost of Electricity (LCOE). NPV assesses the potential profit/loss of a project by considering the time value of the projected cash flows over the project’s lifetime. Positive NPVs are desired by investors since a profitable investment option is expected when the value of money is applied to the analysis. Second, LCOE represents the total lifetime cost of a power generation project, considering the time value of both costs and projected energy generation. Lower LCOE values are preferred because they indicate higher cost-effectiveness for the project. The complete mathematical formulation underlying the techno-economic analysis is presented in Eq. (7) through Eq. (10).

| $R_{t}=\sum_{i=1}^{8760} \left( E_{irr, i}\cdot0.02782+ \left( E_{hh, i}+E_{export, t}\cdot\left( 1-\alpha\right) \right)\cdot\mathrm{PML}_{i}\cdot\mu+E_{del, i}\cdot\mathrm{CEL} \right)_{t}$ | Eq. (7) |
| --- | --- |
| $E_{del, i}=\frac{E_{irr, i}+E_{hh, i}+E_{export, i}\cdot\left( 1-\alpha\right)}{1000}$ | Eq. (8) |
| $NPV=-\left( \frac{INV\cdot\left( 1+\left( 1+r \right)^{10} \right)}{\left( 1+r \right)^{20}}+\mathrm{CAPEX}_{t=0} \right)+\sum_{t=1}^{T} \left( \frac{R_{t}-\mathrm{OPEX}_{t}}{\left( 1+r \right)^{t}} \right)$ | Eq. (9) |
| $LCOE= \frac{\mathrm{CAPEX}_{t=0}+\sum_{t=1}^{30} \frac{\mathrm{OPEX}_{t}}{\left( 1+r \right)^{t}}+ INV\cdot\left( \frac{1+\left( 1+r \right)^{10}}{\left( 1+r \right)^{20}} \right)}{\sum_{t=1}^{30} \frac{E_{del, i}}{\left( 1+r \right)^{t}}}$ | Eq. (10) |

Where $R_{t}$ is the annual revenue derived from the aggregated hourly revenue profile (MXN); $i$ and $t$ are the hour and year counters, respectively; $E_{irr}$, $E_{hh}$, and $E_{export}$ are the energy allocated to irrigation, local households in SAN, and surplus PV energy exported to the grid (Wh), respectively; 0.00278 is the unit energy price for pumping irrigation water (MXN/Wh); $\alpha$ is the curtailment factor applied to energy exported to the grid, set to zero in the baseline scenario; PML is the local marginal price based on the wholesale electricity market serving the SAN area (MXN/Wh); $E_{del}$ is the total delivered energy across irrigation, households, and grid export (MWh); CEL denotes the price of clean energy certificates, set at 20.57 USD/MWh (equivalent to 363.9 MXN/MWh); μ is the energy price multiplier, set at 1 in the baseline scenario; $\mathrm{CAPEX}_{t=0}$ is the required capital investment at the construction phase (year 2022) based on the capacity of the AV system (MXN/kW); $\mathrm{INV}$ is the inverter cost, which is assumed to be replaced in years 10 and 20 (MXN/kW); $\mathrm{OPEX}$ is the annual funding required to cover AV systems' OPEX (MXN/(kW‧year)) and $r$ is the discount rate set at 6.5% as the average of previously executed PV projects in Mexico [8].

The sensitivity analysis was conducted by applying a ±25% perturbation to each parameter relative to the baseline scenario. Inverter cost was excluded from this analysis, given its limited role in the overall CAPEX. The baseline scenario values, as well as probability distribution functions and corresponding cost ranges used in the Monte Carlo simulation, are presented in Table S 4.

Table S 4. The breakdown of cost and revenue elements for techno-economic analysis, sensitivity analysis, and Monte Carlo Simulation (The MXN values are calculated based on USD values obtained from [5]).

| **Item** | | **Baseline** | **Monte Carlo Simulation** | | | |
| --- | --- | --- | --- | --- | --- | --- |
| **Type** | **Breakdown** | **Mexico** | **Max** | **Avg** | **Min** | **Distribution** |
| Cost | CAPEX  (MXN/kW) | 18,574.5 | 22,201.0 | 14,900.3 | 11,657.7 | PERT  (Max, Min, Avg) |
|  | Inverter Cost (MXN/kW) | 973.0 | 2,216.6 | 1,342.7 | 521.9 | PERT  (Max, Min, Avg) |
|  | OPEX  (MXN/kW‧year) | 133.7 | 245.4 | 133.7 | 63.7 | PERT  (Max, Min, Avg) |
| Revenue | CEL  (MXN/MWh) | 363.9 | 454.8 | - | 255.3 | Random  (Max, Min) |
|  | Energy Price Multiplier  (µ) | 1 | 1.25 | - | 0.75 | Random  (Max, Min) |
|  | Curtailment factor  (α) | 0 | 0.25 | - | 0 | Random  (Max, Min) |
|  | Irrigation Energy Price  (MXN/kWh) | 2.78 | 3.48 |  | 2.08 | Random  (Max, Min) |
| Finance | Discount Rate (%) | 6.5 | 7.6 | - | 4.6 | Random  (Max, Min) |

1. **Farm Economics**

Table S 5 presents the price and yield data used for the farm-level economic estimations.

Table S 5. Yield, price, and profit margin values are used for farm economics calculations.

| **Crop Type** | **Yield** | | **Price** | | |
| --- | --- | --- | --- | --- | --- |
|  | **Irrigated** | **Rainfed** | **Irrigated** | **Rainfed** | **Reference Location** |
| Beans | 1.08 | 0.98 | 11660 | 11764 | SAN |
| Green Barley Forage | 18.20 | 11.6 | 750 | 757 | SAN |
| Green Broad Beans | 3.90 | 3.6 | 6210 | 7402 | SAN |
| Maize Grain | 4.70  (3.38 in BAU) | 2.76 | 5893 | 6286 | Tlaxcala/SAN |
| Vetch | 22.23 | - | 522 | - | Tlaxcala |
| Tomato | 98.35 | - | 7425 | - | Tlaxcala |
| Potato | 36.63 | 18.25 | 4224 | 4695 | Tlaxcala |
| Spinach | 18.97 | 18.4 | 5002 | 5000 | Tlaxcala |
| Lettuce | 26.83 | - | 4654 | - | Tlaxcala |
| * The values apply to 50% of the acreage. The yields for crops under the AV canopy are modified based on the yield reduction estimations. | | | | | |

**References**

[1] SIAP. Anuario Estadístico de la Producción Agrícola [Online] Available: <https://nube.agricultura.gob.mx/cierre_agricola/>

[2] N. Taylor *et al.*, "Photovoltaics Geographical Information System: Status Report 2024," 2025.

[3] A. Garrod, S. N. Hussain, and A. Ghosh, "The technical and economic potential for crop based agrivoltaics in the United Kingdom," *Solar Energy,* vol. 277, 2024, doi: 10.1016/j.solener.2024.112744.

[4] S. Rasooli, C. Casiano-Flores, S. Farhoodi, B. Daher, P. A. Cervantes-Aviles, and C. A. Huerta-Aguilar, "Synergetic Benefits of Agricultural Sewage Reuse and Floating Photovoltaics in Mexican Wastewater System: A Municipal-Level WEF Nexus Study," *Glob Chall,* vol. 10, no. 1, p. e00440, Jan 2026, doi: 10.1002/gch2.202500440.

[5] IRENA, "Renewable power generation costs in 2023," Abu Dhabi, 2024.

[6] SIM. Precios de Energía y Servicios Conexos MTR [Online] Available: <https://www.cenace.gob.mx/Paginas/SIM/Reportes/PreEnerServConMTR.aspx>

[7] S. Rasooli, S. Farhoodi, C. Spataru, M. Sandoval-Reyes, P. A. Cervantes-Avilés, and C. A. Huerta-Aguilar, "Techno-economic assessment of floating photovoltaic systems to strengthen local energy security: A case study of the Valsequillo Dam, Mexico," *Energy for Sustainable Development,* vol. 89, 2025, doi: 10.1016/j.esd.2025.101883.

[8] K. S. Bhaskar, K. Raghunathan, and E. Bozkir, "Price of Solar PV Electricity in Developing Countries," 2016.
